# Minimal residual disease in multiple myeloma: current status

**DOI:** 10.1186/s40364-021-00328-2

**Published:** 2021-10-14

**Authors:** Hong Ding, Juan Xu, Zhimei Lin, Jingcao Huang, Fangfang Wang, Yan Yang, Yushan Cui, Hongmei Luo, Yuhan Gao, Xinyu Zhai, Weicui Pang, Li Zhang, Yuhuan Zheng

**Affiliations:** 1 Department of Hematology, West China Hospital, and State Key Laboratory of Biotherapy and Cancer Center, Sichuan University, #37 GuoXue Xiang Street, Chengdu, China; 2 Department of Hematology, The Affiliated Hospital of Chengdu University, Chengdu, China

**Keywords:** Multiple myeloma, Minimal residual disease, Biology, Omics, Gene expression

## Abstract

Multiple myeloma (MM) is a treatable plasma cell cancer with no cure. Clinical evidence shows that the status of minimal residual disease (MRD) after treatment is an independent prognostic factor of MM. MRD indicates the depth of post-therapeutic remission. In this review article, we outlined the major clinical trials that have determined the prognostic value of MRD in MM. We also reviewed different methods that were used for MM MRD assessment. Most important, we reviewed our current understanding of MM MRD biology. MRD studies strongly indicate that MRD is not a uniform declination of whole MM tumor population. Rather, MM MRD exhibits unique signatures of cytogenetic aberration and gene expression profiles, unlike those of MM cells before therapy. Diagnostic high-risk MM and low-risk MM exhibited a diversity of MRD features. Clonal evaluation may occur at the MRD stage in MM. The dynamics from the diagnostic MM to MRD correlate with the disease prognosis. Lastly, on the aspect of omics, we performed data-based analysis to address the biological features underlying the course of diagnostic-to-MRD MM. To summarize, the MRD stage of disease represents a critical step in MM pathogenesis and progression. Demonstration of MM MRD biology should help us to deal with the curative difficulties.

**Supplementary Information:**

The online version contains supplementary material available at 10.1186/s40364-021-00328-2.

## Background

Multiple myeloma (MM) is a hematological cancer characterized by malignant plasma cells accumulation in bone marrow [1]. In the past few decades, the use of autologous stem cell transplantation (ASCT), proteasome inhibitors, and immunomodulatory drugs have revolutionarily extended MM patients’ overall survival (OS) [2–5]. More recently, the approval of novel agents, such as CD38 targeting antibodies and XPO1 inhibitors, in MM clinics give patients additional beneficial options [6–10]. However, even with the significant improvement of disease management, MM remains incurable. Most MM patients, if not all of them, relapse after treatment [11]. Multiple rounds of therapies and relapse lead to refractory disease and patients lost.

More and more MM patients have achieved deep remission and good prognosis through the use of novel drugs and combination therapies. Such new trend in MM treatment calls for update of treatment efficacy evaluation systems [12]. For example, the current guideline for complete remission (CR) in MM includes “negative immunofixation on the serum and urine, disappearance of soft tissue plasmacytomase, and less than 5% plasma cells in bone marrow aspirates” [13]. With such CR assessment, although more than half of newly diagnosed MM patients can achieve CR, approximately 68% relapsed within 2 years [14]. In order to further improve the efficiency of disease evaluation, IMWG included the minimal residual disease (MRD) assessment as an additional response evaluation in 2016 [15].

Minimal residual disease (MRD) refers to a small number of cancer cells surviving after treatment. As early as the late 1980s, the term MRD was introduced in MM clinics to evaluate therapeutic efficacy [16]. Historically, MM MRD was detected by immunohistochemistry, a method that could just provide a vague measurement of residual plasma cells in patients’ bone marrow (BM) biopsies [17]. In 1993, a British group reported PCR-based MRD examination in MM patients after allogeneic bone marrow transplantation [18]. In 1999, immunophenotyping flow cytometry was applied for MM MRD detection [19]. According to the International Myeloma Working Group (IMWG) definition in 2016, MM MRD is the persistence or re-emergence of very low levels of cancer cells in complete remission (CR) patients with about 1 tumor cell in at least 10^5^ normal BM cells [15]. Advances in technique aim to provide highly sensitive methods for MRD assessment.

The clinical implication of MM MRD has long been recognized; persistent MRD after treatment indicates that the tumor cells are not completely eradicated and a relapse in the near future is expected. In 2008, a Spanish team showed that after ASCT, MRD^+^ MM patients had inferior progression-free survival (PFS) and OS compared with MRD^−^ patients [20]. Until now, independent studies all show that MRD negativity is a superior prognostic factor in various MM treatment regimens [21–23]. To our surprise, with hundreds of articles pertaining to MM MRD studies, less than five publications investigated MM MRD biology, such as mutations, cytogenetic aberration, gene expression signature, and cell signaling. Hereby, with brief review of MM MRD clinical significance and detection methods, we mainly focused on our current understanding of MM MRD biology. With the limited but pioneering data, we provided an overview of MM MRD biological characteristics.

## Methods for myeloma MRD assessment

The aim of MRD assessment is to detect residual tumor cells with high sensitivity. At present, there is no standard method for MRD detection. IMWG recommends either intramedullary or extramedullary MRD detection [15]. For intramedullary MRD detection, the cells from patients’ bone marrow aspirations are subjected to different detection methods, including allele-specific oligonulceotide quantitative polymerase chain reaction (ASO-qPCR), multi-parameteric flow cytometry (MFC), next-generation flow cytometry (NGF), or next-generation sequencing (NGS) [24–36]. According to IMWG’s recommendation in 2016, MRD negativity is no cancer cells being detected in the background of at least 10^5^ normal cells by MFC or NGS technology in MM patients who have achieved CR. Alternatively, extramedullary MRD detection is mainly based on imaging techniques, such as positron emission tomography with computed tomography using ^18^F-deoxyglucose (FEG-PET/CT) or magnetic resonance imaging (MRI) [37–40]. The pros and cons of each MRD detection method are summarized in Table 1. In general, for assessment sensitivities, NGS or NGF > MFC > ASO-qPCR, while for applicability, MFC or NGF > NGS > ASO-qPCR. ASO-qPCR requires diagnostic samples to identify patient-specific clonotype sequences for primer designation. Therefore, ASO-qPCR has low applicability.

**Table 1:** Advantages and disadvantages of MRD detection methods

Techniques	Sensitivity	Advantages	Disadvantages
MFC --- Identification of MM cells by Flow cytometry analysis of MM-specific cell surface antigens.	10^− 4^ (4–6 colour) 10^− 5^ (8–10 colour)	1) Strong applicability (90–100%) 2) Fast, economical and efficient; 3) Built-in evaluation of sample quality; 4) Not affected by SHM and clonal evolution.	1) Need to be tested within 24-48 h; 2) Data analysis needs professional knowledge and technology; 3) Complex data visualization; 4) Cannot detect cytogenetic characteristics.
NGF ---Optimized version of MFC with higher sensitivity.	10^−6^	1) Nearly 100% applicability; 2) EuroFlow Consortium standardization; 3) Highly automated; 4) Based on the analysis of large numbers of cells;	1) Need to be tested within 24-48 h; 2) Cannot detect clonal evolution; 3) Complex data analysis.
ASO-qPCR ---Identification of MM cells by amplifying MM-specific immunoglobulin gene rearrangement.	10^−5^	1) Wide range of applications (it can be used in almost all laboratories); 2) Fully standardized detection method and data interpretation standards; 3) No need to process bone marrow samples immediately;	1) The applicability is reduced to 42–75% when using universal primers; 2) The construction of a standard curve will consume a large number of limited bone marrow DNA samples; 3) May provide false negative results.
NGS --- Identification of MM cells by sequencing the IGH/IGK/IGL loci.	10^−6^	1) No need to process samples immediately and no need for a standard curve; 2) Can capture almost all Ig gene rearrangements; 3) Uses consensus primers for clonality detection and subsequent MRD analysis; 4) Adaptive Biotechnologies (Seattle, WA, US) standardization;	1) May be affected by SHM; 2) No sample built-in test; 3) It is time-consuming, labor-intensive, expensive, and cannot be universally used in clinics and laboratories; 4) Interpretation of results is really difficult, requiring high expertise.
FDG-PET/CT --- Identification of MM cells by assessing tumor metabolic aberration	Spatial resolution limit of approximately 5 mm	1) Residual active clonal plasma cells can be detected in residual osteolytic lesions; 2) Accurately maps the sites of bone and extra-medullary disease; 3) It is complementary to cellular or molecular-based techniques;	1) May provide false positive results, for example: recent use of chemotherapy or/and growth factors to induce bone marrow reconstitution; 2) May provide false negative results, for example: lack of hexokinase enzyme or use of high-dose steroids; 3) Significant cost.

*Abbreviations*: *MRD* measurable residual disease, *MFC* multiparameter flow cytometry, *NGF* next-generation flow cytometry, *ASO-qPCR* allele-specific oligonucleotide quantitative polymerase chain reaction, *NGS* next-generation sequencing, *FDG-PET/CT* positron emission tomography with computed tomography using 18F-deoxyglucose

In the past few years, MRD detection techniques have been developed rapidly with great improvements in sensitivity and applicability. NGS is becoming an important method for MRD detection to guide individualized therapy. In NGS-based MRD assessment, the IgH/IgK/IgL loci are sequenced to capture Ig gene re-arrangements in residual MM cells. The NGS data could be further interpreted to identify subclones, clonal evolution, and cloning tides at the MRD stage [24, 25, 41]. Another trend of MM MRD assessment is to combine NGF, NGS, and PET-CT for comprehensive MRD detection [42, 43]. Since MM is focally distributed in the BM, there is a possibility that the lesion tissue is not obtained in the BM aspiration. In addition, some patients may present extramedullary residual plasma cells after treatment. Whole-body imaging, such as PET-CT or MRI, is able to catch those residual diseases. A recent study suggests that whole-body diffusion-weighted MRI (WB-DWI-MRI) may provide better MRD assessment than FDG PET-CT [44]. With availability of such functional imaging techniques, the precise evaluation of response has become feasible also for MM lesions in bones and other organs. Thus, intramedullary MRD negativity, determined by MFC or NGS, plus extramedullary WB-DWI-MRI or PET-CT negativity may provide more accurate assessment for deep remission. Of notice, many new techniques for MRD assessment, such as matrix-assisted laser desorption/ionization mass spectrometry (MALDI-TOF MS) methods [45], liquid chromatography-mass spectrometry (LC-MS) methods [46], circulating cell-free DNA (cfDNA) [47] and single cell RNA-sequencing (scRNA-seq) [48], are currently under investigations at laboratory and pre-clinical stages. Those new techniques may dramatically change MM MRD assessment in future.

## Clinical significance of myeloma MRD

The depth of remission in MM after therapy is closely related to the prognosis of the disease [32]. Therefore, MRD status provides supplementary prognostic stratification in CR MM patients. A series of studies [20, 32, 42, 49–64] showed that MRD negativity was positively associated with prolonged PFS and OS in MM (Table 2). The inclusion criteria of Table 2 data were publications with the key words “minimal residual disease,” “multiple myeloma,” and “overall survival,” while the exclusion criteria were 1) results from meta-analysis and review; 2) study population of less than 100; 3) no survival data; 4) hazard ratios (HRs) were not reported or results were not statistically significant. Based on tight correlation of MRD status of MM treatment outcome, IMWG recommends MRD tests for all MM patients who have achieved CR [15]. The sensitivity of MRD detection affects the prognostic value of MRD [32]. MRD negativity determined by more sensitive methods, such as NGF or NGS, had better prediction of prognosis than that determined by less sensitive methods, such as 4-color MFC [65]. The correlation between MRD and other MM prognostic factors is complicated and calls for more investigation. Newly diagnosed MM is stratified with the Revised International Staging System (R-ISS) as high risk (HR) or standard risk (STR). International Staging System (ISS), based on serum β2-microglobulin and serum albumin, was published on 2005 to stratify MM patients at diagnosis [66]. R-ISS was based on ISS with additions of genetic risk factors and lactate dehydrogenase (LDH) levels to achieve more accurate prognostic stratification of MM patients than ISS [67] . In a retrospective clinical study, the 5-year OS rates in the R-ISS I group, R-ISS II group and R-ISS III group were 82, 62 and 40%, respectively [67]. In general, R-ISS stratified STR-MM has superior OS and PFS compared with HR-MM [42, 65, 68, 69]. However, MRD negativity overcomes HR-conferred MM inferior survival outcome; the time-to-progression (TTP), OS, and PFS of MRD^−^ HR-MM are similar to that of STR-MM [65, 68]. Furthermore, MRD^+^ STR-MM patients obtain better OS than MRD^−^ HR-MM [42, 43, 65, 68]. Lastly, different studies have shown that as long as reaching MRD^−^ status, the treatment arm rarely has an effect on MM PFS [54, 70]. Together, those clinical data may suggest that not only MRD status but also biological features of MRD, such as cytogenetic aberration of MRD, affect the progression of MM at the post-MRD stage. With recognition of the importance of MRD in MM, FDA published a guideline to help sponsors use MRD as a biomarker in clinical trials of new drug applications and promote the marketing of drugs and biological products for the treatment of hematological tumors [71]. Thus, it is suggested to elaborate the significance of MRD positive status beyond MRD negative features.

**Table 2:** Selected clinical studies of MRD in multiple myeloma

Study	MRD technique (sensitivity)	MRD^+^ rate	Survival outcomes (MRD-negative vs MRD-positive)
Paiva et al. 20087 [20] PMID:18669875	4-color MFC (10^− 4^)	58% (*n* = 295)	median PFS: 71 mo vs 37 mo (HR = 0.28, 95%CI 0.17–0.43, *P* < 0.001) median OS: NR vs 89 mo (HR = 0.50, 95%CI 0.38–0.64, *P* = 0.002)
Rawstron et al. 2013 [50] PMID:23733781	6-color MFC (10^− 4^)	38% (*n* = 397)	median PFS: 28.6 mo vs 15.5 mo (HR = 0.55, 95%CI 0.43–0.71, *P* < 0.001) median OS: 80.6 mo vs 59.0 mo (HR = 0.64, 95%CI 0.45–0.91, *P* = 0.018)
Chakraborty et al.2017 [55] PMID:28115277	6- color or 7-color MFC (2 × 10^− 5^–10^− 4^)	44% (*n* = 185)	median PFS: 26 mo vs 17 mo (HR = 0.45, 95%CI 0.31–0.66, *P* < 0.001) median OS: NR vs 50 mo (HR = 0.55, 95%CI 0.32–0.92, *P* = 0.023)
Deng et al.2018 [59] PMID:29779345	MFC (10^− 4^)	55% (*n* = 106)	median PFS: NR vs 17 mo (HR = 0.23, 95%CI 0.09–0.58, *P* < 0.001)
Gu et al.2018 [60] PMID:30142420	MFC (5 × 10^− 5^ -10^− 5^)	64% (*n* = 104)	median TTP:NR vs 26.4 ± 11.5 mo (HR = 0.18, 95%CI 0.08–0.43, *P* < 0.001) median OS:NR vs 40.7 ± 13.7 mo (HR = 0.08, 95%CI 0.02–0.27, *P* < 0.001)
Perrot et al.2018 [70] PMID:30249784	NGS (10^−6^)	62% (*n* = 239)	median PFS: NR vs 20 mo (HR = 0.18, 95%CI 0.12–0.28, *P* < 0.001) 3-year OS: 96% vs 86% (HR = 0.26, 95%CI 0.10–0.68, *p* = 0.008)
Li et al.2019 [61] PMID:30721336	MFC (10^− 4^)	75% (*n* = 123)	median PFS: NR vs 26 mo (HR = 0.29, 95%CI 0.12–0.69, *P* < 0.001) 4-year OS:91.7% vs 66.3% (HR = 0.13, 95%CI 0.02–0.96, *P* = 0.008)
Tschcautsher et al.2019 [62] PMID:30945330	7-color MFC (2 × 10^−5^)	30% (*n* = 460)	median TTNT: 37.6 mo vs 23 mo (HR = 0.51, 95%CI 0.40–0.66, *P* < 0.001)
Alonso et al.2020 [63] PMID:32433744	4-color MFC or NGS (> 10^− 4^)	48% (*n* = 139)	median PFS: 83 mo vs 48 mo (HR = 0.49, 95%CI 0.27–0.86, *P* = 0.011)
Medina et al.2020 [64] PMID: 33127891	NGF (2 × 10^− 6^)	45% (*n* = 106)	3-year PFS: 91.4% vs 50% (HR = 0.20, 95%CI 0.09–0.44, *P* < 0.001) 3-year OS: 96.6% vs 74.9% (HR = 0.18, 95%CI 0.05–0.62, *P* = 0.007)
Paiva et al.2020 [65] PMID: 31770060	NGF (2 × 10^−6^)	43% (*n* = 357)	36-month PFS: 87% vs 50% (HR = 0.21, 95% CI 0.12–0.36, *P* < 0.001) 36-month OS: 96% vs 88% (HR = 0.26, 95% CI 0.10–0.67, *P* = 0.005)

*Abbreviations*: *MRD* measurable residual disease, *MFC* multiparameter flow cytometry, *NGF* next-generation flow, *NGS* next-generation sequencing, *HR* hazard ratio, *CI* confidence interval, *PFS* progression-free survival, *OS* overall survival, *TTNT* time to next treatment, *TTP* time to progression, *mo* months, *NR* not reached

More recently, many clinical trials have included MRD negativity rates as readouts for the measurement of results. To be specific, MRD, one of the standard evaluable endpoints, is a must when designing clinical trials involving both monoclonal antibody-based therapies and transplantation. A number of clinical trials have put MRD as a primary endpoint, and set MRD negativity rate improvement as a surrogate endpoint for MM drug approval [43]. Using MRD as a surrogate endpoint enables researchers to obtain clinical trial results in a relatively short period of time, thereby speeding up the approval of new drugs for MM.

## Mutation and cytogenetic signature of myeloma MRD

The cytogenetic features of MM MRD are still largely unknown. In 2020, a Chinese group reported, for the first time, a cytogenetic study including MM MRD [72]. They retrospectively analyzed 193 MM patients who had at least one cytogenetic abnormality (CA) at diagnosis and achieved PR or above after 4–6 cycles of therapy. Residual plasma cells were detected by MFC, and cytogenetic aberration in plasma cells was detected by interphase fluorescence in situ hybridization (iFISH). According to their data, MM exhibited heterogeneous patterns of cytogenetic dynamics from diagnosis to MRD, including gaining new CAs or some CAs becoming dominant, the unbalanced declination of different pre-existing CAs, the uniform declination of different pre-existing CAs, unchanged CA patterns, and CAs lost (undetectable). Approximately 34% of analyzed patients fell into the first 2 CA dynamics groups, which were considered therapy-induced clone selection, while the others had improved or stable cytogenetics from diagnosis to MRD. The patients without clone selection at the MRD stage had longer TTP than those with clone selection, and such association neglected the impact of HR-CA at the diagnosis. The group did not show whether HR-CA at the MRD stage was associated with inferior patient outcomes. But the patients with undetectable CA in MRD had superior OS. In short, they provided evidences of clone evolution at the MRD stage. The dynamics of cytogenetic alteration from diagnosis to MRD were associated with the patient’ prognosis.

Very recently, the Spanish group published another MM MRD study including both mutation and gene expression profiles [73]. In the study, MRD status was determined by NGF, and the residual plasma cells were sorted for profiling assays afterward. Mutation profiles of diagnostic MM cells and MRD were examined by whole-exome sequencing in 40 paired patients. Like the CA dynamics, their data also suggested that mutations and copy number alteration (CNA) from diagnostic MM to MRD exhibited heterogeneous patterns of change. Diagnostic HR-MM was likely to acquire new mutations in residual plasma cells after treatment, while the actionable mutations, such as KRAS and BRAF, remained persistent at the MRD stage. By contrast, many STR-MM had diminished mutation burden and CNA in MRD cells after treatment. The authors proposed that the genomic instability might contribute to the acquisition of new mutations in HR-MM. Overall, the results supported that diagnostic risk stratification of MM predicts the cytogenetic and mutation features of MRD.

## Gene expression profile of myeloma MRD

Very few studies have investigated the gene expression profiles (GEPs) of MM MRD. In 2015, Paino et al. reported on a study to investigate MM clone heterogeneity [74]. They examined 10 MRD samples by MFC immunophenotyping, and generated 23 marker gene expression profiles by computational calculation. Their work suggested that from the stage of diagnosis to MRD, tumor cells exhibited altered marker gene expression. In 2016, a *Blood* publication reported the first genomic study of MM MRD [68]. MRD was determined by MFC in the study. GEPs of seven paired patients’ plasma cells, diagnostic MM and MRD tumor cells, were examined by microarray. Approximately 1300 genes (fold change 0.1–2.7) were differentially regulated from diagnosis to MRD. Among those genes, the authors showed that activated leukocyte cell adhesion molecule (ALCAM) down-regulation in MRD might contribute to MRD drug resistance. ALCAM is an adhesion molecule interacting with CD6 that mediates intercellular adhesion and migration [75, 76]. Drug treatment induced ALCAM^Low^ RPMI-8226 cells accumulation in vitro. Of note, the patients with ALCAM^Low^ MM had superior OS. Accordingly, they identified the functional genes in MM by MRD cells profiling.

In 2021, another *Blood* publication analyzed diagnostic MM cells and MRD tumor cells using RNA sequencing [73]. MRDs derived from HR-MM (*N* = 14) and STR-MM (*N* = 26) exhibited different GEP signatures and pathway enrichments. From diagnostic plasma cells to MRD tumors, twice as many differentially regulated genes were identified in the STR-MM group than in the HR-MM group. Only a small number of genes, less than 20%, were identified as commonly deregulated genes from diagnosis to MRD in STR-MM versus HR-MM groups. In addition, pathway enrichment analysis suggested that deregulated genes in the STR-MM group versus the HR-MM group fell into different cell signaling pathways. The reactive oxygen species (ROS) pathway was specifically enriched in the HR-MM gene set. Thus, even for patients with the same therapeutic responses, CR, diagnostic HR-MM, and STR-MM may have various patterns of evolution.

**Fig. 1:**
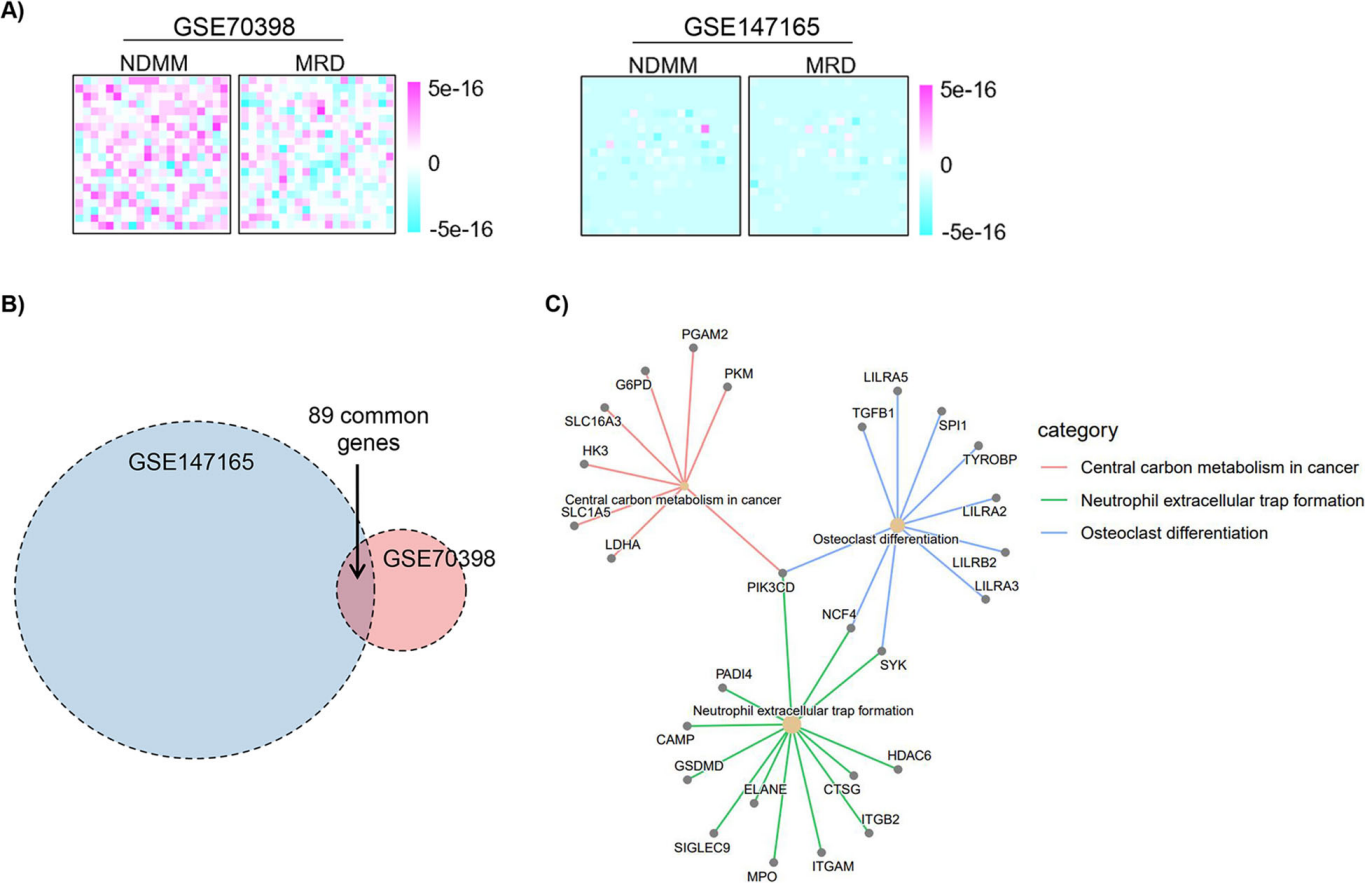
Omics data-based analysis on MRD in multiple myeloma. **A** Dimensionality reduction using GSE70398 (7 paired samples) and GSE147165 (40 paired samples) datasets; **B** Venn diagram, *p* < 0.05, logFC> 1 for both datasets; **C** KEGG pathway enrichment

Both of the above GEP datasets provided us precious raw data on the MRD landscape. Analysis of those data suggested some interesting aspects of MRD regulation, as well as limitations of the data and methodology (data access and analyses are described in Supplementary Information). First, diagnostic MM and MRD MM cells had diversified patterns of gene expression. After dimensionality reduction in both datasets, we found that gene expression values in some MRD modules were diversifying (Fig. 1A). The research data of *Blood* in 2016 [68] showed more intensive changes of gene expression patterns than the data of *Blood* in 2021 [73]. Multiple reasons might cause such a discrepancy. Since the former publication simply included seven paired samples without risk stratification, we don’t know whether the two studies had matched patient characteristics at diagnosis. It’s noteworthy that the patients in the two datasets received different regimens. Patients in the first study received VMP (bortezomib, melphalan, prednisone) or VMP plus Rd. (lenalidomide and dexamethasone) regimens, while patients in the second study received VRD, autologous stem cell transplantation (ASCT), melphalan regimens. Different drugs may induce different selection stress on MM cells, and result in MRDs with different GEP alteration. Lastly, the two studies used disparate methods for gene expression profiling; the former used microarray and the latter used RNA sequencing. Second, differentially regulated genes from diagnosis to MRD in both datasets had merely a small number of common genes (Fig. 1B). We hypothesized that those common genes from the two datasets might contain the core gene alterations of MRD generation and function. Therefore, we performed KEGG pathway enrichment using common differentially regulated genes. The pathways “central carbon metabolism in cancer,” “neutrophil extracellular trap formation,” and “osteoclast differentiation” were enriched from diagnosis to MRD (Fig. 1C). Those pathways indicated the potential roles of cell metabolism and tumor microenvironment in MRD development. Further evidences is needed to verify the results.

## Future perspective

Growing evidence suggests that MRD evaluation provides more accurate prognostic assessment than conventional response-based evaluation in MM. The clinical significance of MRD indicates the importance of MRD biology in MM progression. The studies we reviewed in this article further suggest that the dynamics from diagnosis to MRD also harbor clinical significance. MRD cells are resistant tumor cells that survived anti-MM therapy. How the MRD cells escape the treatment is still largely unknown. Clone selection and evolution occur at the MRD stage of disease. To a degree, both genetic alteration and transcriptional regulation contribute to MRD drug resistance. Demonstration of MRD biology, especially for HR-MM-derived MRD, is an urgent need to reveal MRD’s drug resistance, and a basis for development of MRD-targeting therapy. Furthermore, it may be interesting to examine MRDs from different therapies, whether those MRDs share the same genetic characteristics and GEPs. The answers may indicate the mechanism to screen out MRD. In addition, the ratios of MRD positivity after different therapies may indicate the capability of different drugs in screening out resistant MRD cells. Lastly, investigation on the molecular basis of the post-negative-MRD stage causing MM progression is another interesting topic. How do the residual tumor cells behave after the termination of stringent treatment? What is the appropriate way to eradicate residual tumor cells, and when is the appropriate time?

There are many difficulties to carrying out basic research on MRD in MM. First, the number of MRD tumor cells is too low to be investigated by most experimental methods. Ethical considerations prohibit large-scale collection of MRD cells from MM patients. And findings from limited MRD omics data may be influenced by bias and false results. Second, MM lack in vitro and in vivo research models for MRD studies. MRD is a small population of resistant MM cells that survived after therapy-induced clone selection in patients. The heterogeneous nature of MRD can hardly be simulated by currently used MM cell lines and MM mouse models. In particular, the dynamics of diagnostic MM to MRD cannot be reproduced in an experimental setting. Third, the strategy of MRD-related gene study may need reconsideration. A gain-and-loss model is widely used to investigate a gene’s function in cancer cell lines and animal models. In addition, the correlation of a gene’s expression, usually between the stages of diagnosis and prognosis, is a strong indicator that the gene plays a certain role in MM pathogenesis or therapeutic response. However, as our understanding of MRD in MM is at the very beginning, we don’t know yet whether all critical players at the MRD stage of the disease exhibit constant function during the whole disease course. MM cell lines based on gain-and-loss experiments may not reflect the real regulation within the residual tumor cells.

## Conclusions

MRD represents a critical stage in MM treatment, during which the patient has a minimal number of resistant tumor cells. Both MRD status and the dynamics of diagnosis-to-MRD transition have a prognostic value for MM. A significant amount of gene expressions and signaling pathways are altered within the tumor cells from diagnosis to MRD. Each individual may yield discrepant dynamic patterns of diagnosis-to-MRD transition. Clone evolution may occur at the MRD stage in MM and is associated with inferior long-term outcomes. Diagnostic risk stratification affects the MRD stage of disease; high-risk and standard-risk MM-derived MRDs may exert a wide variety of gene expression profiles.

Many questions about MRD remain unanswered. More exploratory experiments will help a lot to demonstrate MM MRD.

## Supplementary Information

**Additional file 1.**

## Data Availability

Not applicable.

## Abbreviations

MM: Multiple myeloma
MRD: Minimal residual disease
ASCT: Autologous stem cell transplantation
OS: Overall survival
BM: Bone marrow
IMWG: International Myeloma Working Group
CR: Complete remission
PFS: Progression-free survival
MFC: Multi-parameteric flow cytometry
NGF: Next-generation flow cytometry
ASO-qPCR: Allele-specific oligonucleotide quantitative polymerase chain reaction
NGS: Next-generation sequencing
FDG-PET/CT: Positron emission tomography with computed tomography using ^18^F-deoxyglucose
MRI: Magnetic resonance imaging
R-ISS: Revised International Staging System
HR: High risk
STR: Standard risk
TTP: Time-to-progression
CA: Cytogenetic abnormality
iFISH: Interphase fluorescence in situ hybridization
CAN: Copy number alteration
GEPs: Gene expression profiles
ALCAM: Activated leukocyte cell adhesion molecule
ROS: Reactive oxygen species
VMP: Bortezomib, melphalan, prednisone
Rd: Lenalidomide and dexamethasone
HR: Hazard ratio
CI: Confidence interval
TTNT: Time to next treatment
mo: Months
NR: Not reached
