# Supplementary material for: Minimal residual disease in multiple myeloma: current status
Source: Biomark Res. 2021 Oct 14;9:75. doi: 10.1186/s40364-021-00328-2 (PMC8515655; doi:10.1186/s40364-021-00328-2)
Supplement: Supplementary file 1 — Additional file 1. [file 40364_2021_328_MOESM1_ESM.docx]

**Supplementary Information**

**Gene Expression Profiles Data Access**

Gene expression profiles (GEP) GSE70398 and GSE147165 were downloaded from the Gene Expression Omnibus database (GEO; http://www.ncbi.nlm.nih.gov/geo/) database. GSE70398

was based on the GEO platform, GPL6244[HuGene-1_0-st] Affymetrix Human Gene 1.0 ST Array [transcript (gene) version], while GSE147165 was based on the GEO platform, GPL18573 Illumina NextSeq 500 (Homo sapiens). GSE70398 included 7 patient-paired samples, while GSE147165 included 40 patient-paired samples. In both datasets, diagnostic MM was control group and MRD clonal PCs was case group.

**GEP Data Analyses**

Raw data were processed with R language. Since GSE70398 and GSE147165 datasets were generated by microarray and RNA sequencing, respectively, different analytic methods were applied for data processing and analysis. For GSE70398, according to GPL6244 platform annotation information, all probe IDs were transformed into gene symbols. When a symbol corresponds to more than one probe, the maximum value of gene expression is obtained. After that, scale function and melt function of reshape2 r package are used to preprocess and standardize all gene expression values. Then test the data quality by hierarchical clustering analysis and principal component analysis. Linear models for analysis package (http://www.bioconductor.org/packages/release/bioc/html/limma.html) [[1](#_ENREF_1)]were used to screen the differentially expressed genes (DEGs) between MRD clonal PCs (case group) and diagnostic MM (control group). *p*<0.05 and log FC (fold change)>1 were set as the thresholds. For GSE147165, first, we constructed the dds matrix, including expression matrix, sample information matrix and difference comparison matrix. Counts table were read as the expression matrix and sort the sample processing information as the sample information matrix. The difference expression matrix defines the control group and the experimental group. We used DESeq2 package to identify DE genes. After that, we standardized the dds matrix and used the result function to get the result, and then used the "org.Hs.eg.db" package to annotate the gene. We Compared the data of the control group and the experimental group, and conducted a difference analysis to screen the data with statistical significance (*p*<0.05).

The Kyoto Encyclopedia of Genes and Genomes (KEGG; http://www.genome.ad.jp/kegg) was a database applied for conducting pathway analysis for genes or other molecules.[[2](#_ENREF_2)] KEGG pathway enrichment analysis were performed using common DEGs from above 2 datasets, with the threshold set at *p*<0.05**.**

**References**

1. Ritchie ME, Phipson B, Wu D, Hu Y, Law CW, Shi W, et al. limma powers differential expression analyses for RNA-sequencing and microarray studies. Nucleic Acids Res. 2015;43(7):e47.<http://doi.org/10.1093/nar/gkv007>

2. Kanehisa M, Goto S. KEGG: kyoto encyclopedia of genes and genomes. Nucleic Acids Res. 2000;28(1):27-30.<http://doi.org/10.1093/nar/28.1.27>
